# Limiting Severe Outcomes and Impact on Intensive Care Units of Moderate-Intermediate 2009 Pandemic Influenza: Role of Infectious Diseases Units

**DOI:** 10.1371/journal.pone.0042940

**Published:** 2012-08-14

**Authors:** Sergio Carbonara, Giuseppe Bruno, Giuseppe Di Ciaula, Anna Donata Pantaleo, Gioacchino Angarano, Laura Monno

**Affiliations:** Clinic of Infectious Diseases, School of Medicine and Surgery, University of Bari, Bari, Italy; Naval Medical Research Unit 6, United States of America

## Abstract

**Purpose:**

The rate of severe outcomes of patients with 2009 pandemic (A/H1N1) influenza (2009pI) hospitalized in non-intensive care units (ICUs) has not been defined thus far. This study aims to assess the efficacy of the management of patients with influenza-like illness (ILI) of moderate intermediate severity in an infectious diseases unit (IDU) during the first wave of 2009pI and its influence on the burden of ICUs.

**Methods:**

All patients hospitalized from October 27, 2009, to February 5, 2010, with ILI were included in this prospective observational study. The IDU was organized and the staff was trained to provide intermediate care; patients were transferred to the ICU only if they required invasive ventilation, extracorporeal membrane oxygenation, or advanced cardiovascular support. Demographic data, clinical presentation, coexisting medical conditions, and laboratory and radiological findings were recorded and analyzed, as well as treatment and outcome data.

**Results:**

Overall, 108 patients (median age 36 years [IQR 27–54], 57.4% males) including 66.7% with ≥1 risk factor for severe influenza, 47.2% with confirmed 2009pI by RT-PCR and 63.9% with pneumonia, were enrolled in the study. All subjects received intravenous fluids and 83.3% were administered oseltamivir, 96.3% antibacterials, 19.4% oxygen therapy without ventilatory support, and 10.2% non-invasive ventilation. A total of 106 (98.1%) subjects were discharged after a 6-day median hospital stay [IQR 4–9]. Two patients (1.9%) were transferred to the ICU. There were no deaths.

**Conclusions:**

These results suggest that the aggressive treatment of patients with moderate intermediate severity 2009 pandemic ILI in non-ICU wards may result in a low rate of severe outcomes and brief hospitalization. IDUs, if properly organized for intermediate care, may efficiently provide correct disease management, in addition to complying with infection control requirements, thus reducing the burden of the pandemic on ICUs. Further studies are warranted to evaluate the outcome of patients with moderate intermediate 2009pI in different non-ICU settings.

## Introduction

Despite its rapid spread among the population worldwide, the 2009pI was characterized by overall moderate severity, with higher rates of asymptomatic and mild cases in comparison to previous pandemics and several interpandemic influenza outbreaks [1]. Notwithstanding, a remarkable burden of patients with differing grades of clinical severity resulted, seriously challenging health services, including hospitals and, in particular, intensive care units (ICUs) [2], [3]. Low infection rates occurred among the elderly population [1], [4], and consequently most hospitalized patients, including those with severe outcomes (e.g., ICU admission and death), were young and middle-aged adults [1], [3]–[8], although a correlation between increasing severity and age was evident. According to the European Centre for Disease Prevention and Control (ECDC), around 80% of deaths in Europe occurred in patients under the age of 65 years [1]; in the USA, approximately 90% of estimated hospitalizations and 87% of estimated deaths occurred in people younger than age 65 [7]. Worldwide, the median age was 19, 42 and 46 years among hospitalized patients, those admitted to ICUs, and fatal cases, respectively [4]. The risk of death in hospitalized patients, however, was highest in those older than 64 years [4], [7]. Furthermore, the risk for complications and severe outcomes was associated with the presence of chronic diseases or other factors such as obesity and pregnancy [4], [8]. Nonetheless, underlying risk conditions were not detected in 28–69% of patients hospitalized in non-ICUs [4], [5], [8], and in 22–48% of those who either were admitted to the ICU or died [4], [5], [8], [9]; ECDC reports that around 25–30% of deaths attributed to the pandemic in Europe were in entirely healthy young adults and outside the traditional risk groups [1].

Several large surveillance studies from differing global areas, conducted among hospitalized patients during the first wave of 2009pI [3]–[5], [8], [10]–[16], have described the risk factors for severe outcomes, reporting an ICU admission rate of 14–27% and an overall mortality of 4–6%. However, to our knowledge, the rate of severe outcomes among 2009pI patients admitted to medical units has never been defined, as the above studies do not distinguish between those patients directly admitted to ICUs after presentation to hospital or emergency services from those transferred to the ICUs following a period in non-ICU wards; in addition, the mortality rate was not specified for each setting.

The present study aims to describe the outcomes and impact on ICUs of patients with moderate-intermediate severity influenza-like illness (ILI) who were hospitalized during the first wave of 2009pI in an infectious diseases unit (IDU) capable of providing an intermediate-level of care, along with the necessary measures for infection control, and specialized management of the infection and its complications.

## Methods

### Ethics Statement

Each patient provided informed written consent for the collection, recording, analysis and publication of data for the present study. The study was sent to the hospital Ethical Committee, which, according to Italian regulations regarding observational studies [17], is not required to provide formal ethical approval.

The clinic of Infectious Diseases of the University of Bari is located in the largest hospital (1.550 beds) of the Apulia Region of Southern Italy. The inpatient unit of the clinic is composed of 22 beds served continuously by six infectious diseases specialists, and is designated by the regional pandemic plan as a reference institution for the hospital care of ILI patients requiring a moderate to intermediate level of care; this regional plan provides for limiting admission to ICUs to those patients requiring invasive mechanical ventilation, extracorporeal membrane oxygenation (ECMO) or advanced cardiovascular support. The above role assigned to our clinic was motivated both by the specialized competence of this unit for management of infectious diseases, and its compliance with the structural and organizational standards recommended to ensure adequate infection control. These structural characteristics included the availability of 14 beds located in eight airborne infection isolation rooms. Furthermore, the IDU was specifically organized and the staff trained to provide intermediate care for influenza complications, including the assessment of need for emergency oxygen supplementation and non-invasive ventilation (NIV) support, as well as administration, monitoring and discontinuation of these measures. At the start of the pandemic season, the nurse:patient ratio of the unit was increased from the usual 1∶5.5 to 1∶3 in the areas dedicated to intermediate care. When necessary, an intensive-care specialist consultation and transfer to ICUs were readily available.

All consecutive patients hospitalized with an ILI diagnosis from October 27, 2009, to February 5, 2010, (date of the last patient admission during the 2009–2010 pandemic influenza season) were enrolled in this prospective observational study. Diagnosis was based on the following criteria for the definition of ILI established by the Italian Ministry of Health [18]: acute respiratory disease with an abrupt onset with fever ≥38°C, at least one respiratory symptom (among cough, pharyngodynia, nasal congestion) and at least one of the following symptoms: headache, general malaise, asthenia, chills, sweats. At admission, the following were obtained from all patients: chest radiographs, assessment of vital signs including pulse oximetry, supplemented when necessary by arterial blood gas (ABG) measurements, and nose and throat swabs for presence of the 2009pI virus by real-time reverse-transcriptase polymerase chain reaction (RT-PCR) [19], [20]. All patients were asked to undergo blood and sputum cultures before initiating antibiotics. Tests for *Legionella pneumophila* and *Streptococcus pneumoniae* urinary antigens were carried out in patients with pneumonia. Other microbiological investigations were performed on an individual basis according to specific clinical findings. All women of reproductive age who did not declare ongoing menses or pregnancy underwent a quantitative human chorionic gonadotropin blood test. Each patient provided an informed written consent for the collection, recording and analysis of data for the present study, which included the following: demographic information, body-mass index (BMI), cigarette and alcohol use, influenza vaccination, underlying medical conditions, immunosuppressive therapies prior to admission, symptoms related to the current hospitalization and their onset, clinical signs, chest radiograph and other imaging techniques, laboratory tests, antiviral and antibacterial treatments, steroid and other concomitant therapies, oxygen supplementation, NIV and cardiovascular support. Outcomes were classified as either hospital discharge, transfer to ICU or death; hospital length-of-stay was also recorded.

### Definitions

“Moderate-Intermediate disease” was defined as the presence of any of the following: arterial blood pH<7.35 or >7.45, respiratory rate >25 breaths/min or oxygen saturation <94% or arterial partial pressure of oxygen <8 Kpa when breathing room air, heart rate >110 beats/min, white blood cells count <4,000/µL or >12,000/µL, other evidence of organ dysfunction for which hospitalization was required. Within this category, we distinguished patients with moderate from those with intermediate severity based on the level of care required. In particular, “Moderate disease” was defined as an illness requiring a normal ward care only, whereas “Intermediate disease” was defined as the presence of serious organ dysfunction which, although not necessitating ICU admission, required higher care level (“intermediate care”) than the normal ward care. This higher level of care included: prolonged high-flow oxygen supplement, NIV, intravenous drugs for inotropic/vasopressor support or to control cardiac arrhythmias, close monitoring of the patient (continuous non-invasive polyfunctional monitoring of vital parameters, continuous video-monitoring of the patient bed, a minimum of hourly patient observation), frequent assessment (>2 times daily) of laboratory parameters (e.g., ABG, cardiac enzymes, hepatic or renal function tests), chest drainage as required.

“Severe outcome” was defined as ICU admission or death, and “Severe disease” as a critical illness with a severe outcome.

“Risk factors for severe influenza” were defined as any chronic medical condition such as pulmonary, cardiovascular, renal, hepatic, neuromuscular, hematologic, and metabolic disorders; malignancies, immunodeficiency; elderly (aged >65 years); pregnancy, obesity (BMI≥30) [3]–[5], [8], [15], [21]–[23].

According to the IDSA (Infectious Diseases Society of America) and BTS (British Thoracic Society) criteria [24], [25], “Pneumonia” was defined as a pulmonary infiltrate demonstrated by chest radiograph or other imaging technique, not known to be previously present and for which there was no other explanation than infection, in addition to clinical features consistent with an acute lower respiratory tract infection.

## Results

Overall, 108 patients were included in the analysis. The characteristics of these patients are illustrated in **Tables 1** **,** **2** **,** **3**. In particular, median age was 36 years [IQR 27–54], and 62 subjects (57.4%) were males. Seventy-two patients (66.7%) had ≥1 risk factor for severe influenza. Six women were either pregnant or puerperal; a BMI ≥30 was detected in 19 subjects (17.6%), and at least one chronic co-morbidity was noted in 59 patients (54.6%).

**Table 1:** Epidemiological and clinical characteristics of 108 consecutive patients with influenza-like illness hospitalized in an infectious diseases unit during the 2009/10 wave of 2009 pandemic (A/H1N1) influenza.

Characteristics			Patients	
			#	% 1
**Age** (years)	median (IQR)		36 (27–54)	
	16–30		39	36.1
	31–50		39	36.1
	51–65		17	15.7
	>65		13	12.1
**Sex** (male**)**			62	57.4
**Race**	White		98	90.7
	African		10	9.3
**Smoking**			27	25.0
**Risk factors for severe influenza**	≥1 risk factor		72	66.7
	BMI ≥30		19	17.6
	Age >65		13	12.0
	Pregnancy/puerperium		6	5.6
	Chronic co-morbidities	COPD	15	13.9
		asthma	14	13.0
		cardiovascular disease	19	17.6
		diabetes mellitus	8	7.4
		chronic hepatitis	9	8.3
		chronic renal failure	5	4.6
		cancer	5	4.6
		autoimmune disease	4	3.7
		HIV-infection	2	1.9
		other 3	7	6.5
		≥1 chronic co-morbidity	59	54.6
**Seasonal flu vaccination**			6	5.6
**ILI Symptoms**	Fever ≥38°C		108	100 2
	Asthenia		87	80.6
	Cough	non productive	47	43.5
		productive	32	29.6
		cough, any	79	73.1
	Rhinorrhea		7	6.5
	Myo-Arthralgiae		43	39.8
	Headache		33	30.6
	Pharyngodynia		29	26.9
	Chest ache		25	23.1
	Gastrointestinal symptoms	nausea, vomiting	26	24.1
		diarrhoea	8	7.4
		gastrointestinal, any	26	24.1
**Time from symptom onset to hospitalization** (days)	median (IQR)		3 (2–3)	
**Pneumonia**	Any chest radiograph pattern		69	63.9
	Interstitial pathology		12	17.4 4
	Lobular pathology		57	82.6 4

NOTES:

1 Data refer to percent of total number of patients, unless otherwise specified.

2 Fever ≥38°C was a necessary criteria for defining an influenza-like syndrome and, consequently, for the inclusion in the study.

3 Other comorbidities: neurologic disease (1 patient), hypothyroidism (3 patients), hemoglobinopathy (2 patients), glucose-6-phosphate dehydrogenase deficiency (1 patient).

4 Percentage is calculated using the total number of pneumonia patients as denominator.

BMI, body mass index; COPD, chronic obstructive pulmonary disease; HIV, human immunodeficiency virus; IQR, interquartile range; RT-PCR, reverse transcription polymerase chain reaction.

**Table 2:** Principal laboratory and microbiological findings of 108 consecutive patients with influenza-like illness hospitalized in an infectious diseases unit during the 2009/10 wave of the 2009 pandemic (A/H1N1) influenza.

Laboratory findings		Patients			
		# evaluable	# with finding	% 1	
**Leukocyte count** (cells/µL)	median (IQR)	108	8,900 (6,110–11,820)		
	<4,000		13	12.0	
	4,000–10,000		40	37.0	
	>10,000		43	39.8	
**Lymphocyte count** (cells/µL)	median (IQR)	108	1,349 (763–1,855)		
	<1,500		57	52.8	
**AST >40 and/or ALT >45** (U/L)		108	27	25.0	
**CRP** (mg/L)	median (IQR)	108	30.2 (22.0–37.5)		
	>10		72	66.7	
**LDH** (UI/L)	median (IQR)	108	160 (135.7–208.2)		
**Positive RT-PCR** for 2009pI virus			51	47.2	
				
**Positive bacteriological investigations**	Blood culture	100	4 2	4.0	
	Sputum culture	27	3 3	11.1	
	*L. pneumophila* urinary antigen	69	1	1.4	

NOTES:

1 Data refer to percent of total number of patients, unless otherwise specified.

2 Positive blood cultures: Methicillin-sensitive *S. aureus* (2 patients), *E. coli* (1 patient), *S. hominis* (1 patient).

3 Sputum cultures positive for *S. haemolyticus* (2 patients), *M. fortuitum* (1 patient).

AST, aspartate transaminase; ALT, alanine transaminase; CRP, C-reactive protein; LDH, lactate dehydrogenase; 2009pI, 2009 pandemic (A/H1N1) influenza.

**Table 3:** Findings of moderate intermediate severity in 108 patients with influenza-like illness during the 2009/10 wave of the 2009 pandemic (A/H1N1) influenza.

Findings		Patients	
		#	% 1
**Respiratory**	Pneumonia	69	63.9
	Pleural effusion	12	11.1
	Exacerbated COPD/Asthma	24	22.2
	Arterial blood pH<7.35 or >7.45	58	53.7
	Respiratory rate >25/min	30	27.8
	Sp02 <94% or PaO2<8 Kpa	22	20.4
**Cardiovascular**	Acute or exacerbated chronic heart failure	7	6.5
	Myo/pericarditis	2	1.9
	Arrhythmia/electrocardiographic alterations	15	13.9
	Severe dehydration	22	20.4
	Hearth rate >110/min	29	26.9
	Blood pressure: systolic <90 mm Hg or diastolic ≤60 mm Hg	17	15.7
	Elevated myocardial enzymes	18	16.7
**Renal**	Acute or exacerbated chronic renal failure/Dyalisis	15	13.9
	Elevated blood urea nitrogen	15	13.9
	Electrolyte imbalance	9	8.3
**Hepatic**	Acute hepatitis or exacerbated chronic liver disease	6	5.6
**Neurological**	Decreased consciousness	5	4.6
**Haematological**	WBC <4,000 or >12,000/µL	31	28.7
	HGB <10 g/dL	10	9.3
**Gastrointestinal**	Severe vomit/Inability to maintain oral intake	13	12.0
**Sepsis**		3	2.8
**≥1 finding of moderate intermediate clinical severity**		100	92.6
**≥1 finding of moderate intermediate clinical severity, or ≥1 risk-factor** 2		105	97.2

NOTES:

1 Data refer to percent of total number of patients

2 Underlying risk factors for severe influenza are specified in Table 1.

COPD, chronic obstructive pulmonary disease; HGB, Haemoglobin; PaO2, partial pressure of oxygen in arterial blood; SpO2, Oxygen saturation; WBC, white blood cells.

Median time from onset of symptoms to IDU admission was 3 days [IQR 2–3]. Pneumonia was radiologically confirmed in 69 cases (63.9%), 12 of whom (17.4%) showed an interstitial pattern while 57 (82.6%) had a lobular pattern. Positive microbiological analyses are reported in **Table 2**. RT-PCR for 2009pI virus performed on nasal and/or pharyngeal swabs resulted positive in 51 (47.2%) subjects. Overall, 16 patients (14.8%) showed a microbiologically confirmed bacterial co-infection. Blood cultures were performed in 100 patients, yielding isolation of methicillin-sensitive *Staphylococcus aureus* in 2 cases, *Escherichia coli* in 1 and *Staphylococcus hominis* in another. Only 27 patients produced sputum samples for culture, three of which were positive (*Staphylococcus haemolyticus* in 2 patients and *Mycobacterium fortuitum* in 1). *L. pneumophila* urine antigen was detected in one patient. No patient resulted positive for *S. pneumoniae* urine antigen. Four of seven patients with urinary tract infection demonstrated a significant growth of *E. coli* from urine. Culture of pharyngeal swabs was positive in the remaining four subjects (*Streptococcus pyogenes* in 2, *S. aureus* in 2).

The findings of clinical severity are illustrated in **Table 3**. A total 100/108 patients (92.6%) met criteria for moderate-intermediate severity, 21 of whom (21.0%) met criteria for intermediate severity; 105/108 subjects (97.2%) had at least one finding of moderate-intermediate severity or at least one risk factor for severe influenza. The remaining three patients (2.8%) were hospitalized only for social reasons.

Therapy with oseltamivir was initiated in 90 patients (83.3%) within 12 hours after admission. Antibacterials were administered to 104 subjects (96.3%) and corticosteroids to one patient only. The antibacterial regimens most frequently used were the following: beta-lactam plus a respiratory fluoroquinolone (27.8%), a macrolide alone (25.0%), a respiratory fluoroquinolone alone (16.7%), and a beta-lactam plus a macrolide (12.0%). Reasons for prescribing antibiotics were one or more of the following: demonstrated bacterial disease; persistence, worsening or relapse of high fever or other relevant ILI clinical signs; pneumonia; exacerbation of chronic obstructive pulmonary disease; other serious clinical conditions in which a bacterial co-infection was deemed possible; immune-deficiency or other relevant risk factors for severe influenza. Median duration of antibiotic therapy was 7 days (IQR 5–9), with no significant difference between patients in whom RT-PCR for 2009pI virus was positive and those remaining.

All patients received hydro-saline intravenous fluids, 21 (19.4%) required oxygen therapy but no ventilatory support, and 11 (10.2%) were administered non-invasive ventilatory support: continuous positive airways pressure (CPAP) in nine cases and bilevel positive airways pressure (BiPAP) in two.

A total of 106 patients (98.1%) were discharged following fever remission and either normalization or marked improvement of the remaining acute alterations detected at presentation. The median hospital length-of-stay was 6 days [IQR 4–9]. There were no deaths. Two female patients (1.9%) were transferred to the ICU after 1 and 10 days, respectively, following admission to the IDU ward; the transfer to ICU was due to development of an acute respiratory distress syndrome necessitating intubation and invasive ventilation in both cases; one patient was also subjected to ECMO. One of these two subjects was 47 years old and showed no risk factor for severe influenza; the second patient, aged 55, had a body mass index of 40 and suffered from COPD.

## Discussion

The 2009pI created a sudden increase in the demand for access to hospital facilities; in particular, the capacity of ICUs to care for critically ill patients was seriously challenged [2], [3], [10], [15], [26], [27]. Several large multi-center studies from different continents have reported that nearly 14–27% of all patients hospitalized during the first 2009pI wave were admitted to ICUs and mortality ranged from 4% to 6% [3]–[5], [8], [10]–[16]. However, these same studies did not specify the rate of transfers to ICUs or the mortality among patients admitted to non-intensive care units; moreover, the frequency of these adverse outcomes is not known for patients admitted to the various specific non-intensive care units (e.g., intermediate-care/high-dependency units, infectious disease clinics, respiratory wards, or other general or specialized medical departments). This information would assist policy makers in determining the suitability of different non-ICU hospital settings to manage moderate to intermediate severity illness, and the role of some of these settings to safely reduce the ICU patient burden and ICU-associated risks and costs.

The present prospective observational study aimed to report the outcome of patients hospitalized in an IDU with moderate-intermediate ILI severity during the initial 2009pI wave. An ulterior objective of our study was to clarify to what extent this type of specialized unit could effectively serve to decrease the 2009pI ICU patient burden, both by limiting the ICU transfer rate of patients initially hospitalized in a medical ward and by providing, when needed, an intermediate-level of care. These roles were assigned to our IDU by the Apulia regional pandemic plan, which limited the admission to ICUs only to patients necessitating invasive ventilation, ECMO or advanced cardiovascular support.

Our results demonstrate an extremely low-rate of severe outcomes, as 98.1% patients were discharged in clinically stable conditions, only 1.9% required an ICU transfer, and no deaths occurred. Furthermore, we registered a brief median-hospital-recovery (6 days [IQR 4–9]). Notably, more than one-half of our patient population showed at least one risk factor for severe influenza, 92.6% at least one criterion of moderate intermediate clinical severity, 63.9% a pneumonia, and 29.6% necessitated either acute oxygen supplement or non-invasive ventilation support. In addition, one of the two ICU-transferred patients had two co-existing risk factors for a severe outcome and was transferred just one day after IDU admission, suggesting that her outcome would not have been influenced by our unit care. Unfortunately, as mentioned above, similar studies providing appropriate outcome data for patients hospitalized in medical units to serve as comparison have yet to be published to our knowledge; the length of hospital stay is the only information reported by a prior study of patients not admitted to an ICU, and reported a result (5-day median [IQR 3–7]) similar to our observation [5]. Our population resembled a vast proportion of subjects hospitalized elsewhere during the pandemic, since median age (36 years) and frequency of risk factors for severe influenza (66.7%) in our patients fell within the range of these features published by other previously mentioned large series of hospitalized subjects, including ICU admissions and deaths (median age 18–51 years; risk-factors 31–74%) [4]–[6], [8].

A total of 104/108 (96.3%) of our patients were treated with antibiotics, although a bacterial co-infection was demonstrated only in 14.8% of cases; this low bacterial yield might have been influenced by the antibiotics often administered to patients before their hospital admission. Indications for empirical antibiotic therapy, specified in the Results section, were consistent with current recommendations for antibacterial use in 2009pI [28], [29]. Other series [3], [6], [30] have reported an extensive use of antibiotics (82–98%) in confirmed 2009 H1N1-infected patients, despite the fact that a bacterial infection was documented only in 3%–8% cases. Nonetheless, we acknowledge that the broad antibiotic prescription or duration in our patients might have been partly unjustified. However, this extensive usage was influenced (in particular during the first wave of 2009pI) by the following issues: i) debates and scarce evidence regarding indications for empirical antibiotic treatment in the 2009pI patients [31]; ii) the major role of bacterial pneumonia as cause of death during prior influenza pandemics [32], [33]; iii) the possibility that bacterial co-infections may occur early in the development of 2009pI severe illness [28] and considerably contribute to severe outcomes [34], [35]. Moreover, only a minority of influenza-related lower respiratory tract infections are defined microbiologically [24]; based on several reports of 2009pI-associated pneumonia, a bacterial co-infection cannot be demonstrated in most [6] or any patient [36], [37], and can remain undiscovered in up to 29% of cases, being identified only post-mortem [38]. Thus, it has been postulated that a low number of reported bacterial pneumonia during 2009pI might reflect the difficulty of documenting a specific bacterial diagnosis rather than an actual low incidence [3], [38]. Finally, the use of macrolides has been favored due to the adjuvant anti-inflammatory and immunomodulatory activity observed for these molecules in respiratory tract infections [39] including community acquired pneumonia (CAP) [40] and seasonal influenza [41]–[45], and proposed for 2009pI as well [46]. Altogether, these considerations call for further studies aiming to improve the indications for empiric antibiotic use in patients hospitalized with pandemic influenza. The median duration of antibacterial treatment in our population (7 days overall) was not shorter in patients with a positive RT-PCR for 2009pI virus, most likely because of the awareness of a possible non-microbiologically evident bacterial co-infection, as discussed above; furthermore, RT-PCR results for 2009pI virus were often available only late in the patient's clinical course due to an extensive laboratory work-load.

A possible limitation of our study is that only 47% of subjects had confirmed 2009pI with RT-PCR on nasal and pharyngeal swabs. This result, however, is consistent with published data regarding the performance of this virological analysis; in fact, de la Tabla et al [47] reported 44% positivity with this procedure for patients with pandemic ILI. On the other hand, the possibility that the ILI cases negative on 2009pI RT-PCR in our population were due to seasonal influenza viruses was negligible, as the pandemic virological surveillance conducted in Italy during the first pandemic season [48] demonstrated that the 2009pI virus was responsible for 95.6% of all confirmed cases of influenza. A portion of our patients might have been infected by non-influenza respiratory viruses; however, this possible bias would not have involved a significant number of the study population when considering the following: a) the study was performed during the overwhelming first wave of 2009pI, b) all patients presented with an acute febrile illness corresponding to ILI-defining criteria, c) the moderate intermediate, rather than mild, clinical severity and d) the adult age of patients. In any case, ILI patients with a non-confirmed 2009pI virus infection were included in this study because we believe that this option better reflects the “in the field” situation of clinical practice in units involved in the management of these patients. As a matter of fact, after the first weeks of the pandemic, international health authorities no longer recommended routine RT-PCR tests for the 2009pI infection among hospitalized subjects, limiting this approach to specific conditions (e.g., critical patients) [49], [50]. The present study was observational, and this represents a further limitation versus a case-control analysis, such as comparing the outcomes of our patients to those of a similar population admitted to a non-IDU/non–ICU medical unit; these types of studies are currently lacking although highly warranted.

Notwithstanding the possible study limitations discussed above, our data suggest that early and aggressive treatment of patients hospitalized for moderate intermediate 2009pI illness with oseltamivir, antibacterials and support measures including, when appropriate, intravenous fluid restoration, emergency oxygen therapy and NIV, may yield a very low rate of severe outcomes in terms of ICU transfer and mortality, with a brief hospital stay. At the same time, our results might indicate that IDUs can contribute to reducing the burden of 2009pI in ICUs, by efficiently caring for patients requiring hospitalization, even when necessitating intermediate care. However, proper organization and staff training are required for IDUs to deliver an intermediate assistance level, including the correct assessment for use of acute oxygen therapy and NIV support, as well as the correct administration, monitoring and discontinuation of these treatments [51]–[53]. Furthermore, intensive-care specialist consultation and transfer to ICU should be readily available to these medical units.

If confirmed by further studies, our observations suggest that implementing similar IDUs or other different specialized medical units, such as those dedicated to intermediate care (high-dependency units, HDUs), might optimize the outcome of patients with moderate-intermediate severity pandemic ILI and limit the burden of these patients on ICUs. However, many hospitals (most hospitals in Italy) are not equipped with HDUs. Consequently, patients hospitalized because of complicated 2009pI requiring intermediate care are admitted either into ICUs, thereby increasing both the utilization of this limited hospital resource and ICU-related risks and costs, or into medical wards which are not adequate to provide the required intermediate-level of care, thus possibly resulting in an excessive and unjustified rate of severe outcomes (e.g., ICU transfer or death), a longer period of hospitalization and, again, higher costs.

Once organized and the staff properly trained, a medical unit suitable to provide both general and intermediate specialized care for influenza may represent a flexible solution to comply with the demand for the appropriate level of hospital care required. For instance, such a medical unit might normally serve as a general-care specialized ward capable, however, of assisting sporadic patients requiring an intermediate-care due to acute seasonal influenza or other infectious diseases; nevertheless, this same medical unit would be ready to be upgraded, either entirely or a partially, to an HDU (e.g., by implementing a higher nurse:patient ratio) during influenza pandemics or any other infectious disease epidemic which might determine a patient burden necessitating an intermediate-level of care. It should be emphasized, however, that our organizational model, although appearing to well work during the 2009pI, could be overwhelmed in case of future pandemics caused by more virulent viral strains, with more cases requiring intensive care; on the other hand, it is this worse scenario which especially encourages investigation and implementation of all possible strategies, including the specialized medical units proposed herein, which would be capable of reducing the burden of critically ill patients admitted to ICUs.

As to which type of medical unit is more suitable to be designated and organized to effectively care for moderate to intermediate 2009pI illness, this obviously depends on the local organization and available resources. Existing non-ICU/non-HDU wards dedicated to managing emergency oxygen use and NIV on a regular basis (for example, respiratory wards, emergency wards, or other critical care areas [51]–[53]) could well serve this purpose. However, it is essential to ensure that these settings are structurally competent to enable infection control measures and that the staff is trained to properly manage serious disease complications; in addition, the availability of an infectious diseases specialist consultation would be appropriate. For these reasons, IDUs may represent an effective and efficient option, as these settings are already capable of specialized management of influenza and comply with both structural and organizational requirements for infection control.
