# Correction: Limiting Severe Outcomes and Impact on Intensive Care Units of Moderate-Intermediate 2009 Pandemic Influenza: Role of Infectious Diseases Units

**DOI:** 10.1371/annotation/87765867-68f9-4d1a-bd0a-a8e2bba4350e

**Published:** 2012-11-30

**Authors:** Sergio Carbonara, Giuseppe Bruno, Giuseppe Di Ciaula, Anna Donata Pantaleo, Gioacchino Angarano, Laura Monno

The term "moderate-intermediate" has been incorrectly printed without a hyphen in multiple places through the article. It should have a hyphen.

