# Correction: Limiting Severe Outcomes and Impact on Intensive Care Units of Moderate-Intermediate 2009 Pandemic Influenza: Role of Infectious Diseases Units

**DOI:** 10.1371/annotation/c2659ade-820b-475d-acfc-2dea13cf20b1

**Published:** 2012-12-03

**Authors:** Sergio Carbonara, Giuseppe Bruno, Giuseppe Di Ciaula, Anna Donata Pantaleo, Gioacchino Angarano, Laura Monno

There is an error in Citation.

The correct Citation is: Carbonara S, Bruno G, Di Ciaula G, Pantaleo AD, Angarano G, et al. (2012) Limiting Severe Outcomes and Impact on Intensive Care Units of Moderate-Intermediate 2009 Pandemic Influenza: Role of Infectious Diseases Units. PLoS ONE 7(8): e42940. doi:10.1371/journal.pone.0042940 

